# Genome-Wide Identification of *JRL* Genes in Moso Bamboo and Their Expression Profiles in Response to Multiple Hormones and Abiotic Stresses

**DOI:** 10.3389/fpls.2021.809666

**Published:** 2022-01-14

**Authors:** Zhijun Zhang, Bin Huang, Jialu Chen, Yang Jiao, Hui Guo, Shenkui Liu, Muthusamy Ramakrishnan, Guoning Qi

**Affiliations:** ^1^State Key Laboratory of Subtropical Silviculture, Zhejiang A&F University, Hangzhou, China; ^2^Zhejiang Provincial Collaborative Innovation Centre for Bamboo Resources and High-Efficiency Utilization, Zhejiang A&F University, Hangzhou, China; ^3^Co-Innovation Center for Sustainable Forestry in Southern China, Nanjing Forestry University, Nanjing, China

**Keywords:** moso bamboo, *JRL* gene family, synteny analysis, abiotic stress, hormone response, subcellular localization, homodimer

## Abstract

Jacalin-related lectins (JRLs) are a new subfamily of plant lectins that has recently been recognized and plays an important role in plant growth, development, and abiotic stress response. Although moso bamboo (*Phyllostachys edulis*) is an economically and industrially important bamboo worldwide, there has been no systematic identification of JRLs in this species. Here, we identified 25 *JRL* genes in moso bamboo, and these genes are unequally distributed among 10 genome scaffolds. Phylogenetic analysis showed that the moso bamboo JRLs were clustered into four JRL subgroups: I, II, V, and VII. Numerous stress-responsive and hormone-regulated *cis*-elements were detected in the upstream promoter regions of the JRLs. Genome collinearity analyses showed that the *JRL* genes of moso bamboo are more closely related to those of *Brachypodium distachyon* than to those of *Oryza sativa* and *Zea mays*. Sixty-four percent of the *PeJRL* genes are present as segmental and tandem duplicates. qRT-PCR expression analysis showed that *JRL* genes in the same subgroup were significantly downregulated in response to salicylic acid (SA), abscisic acid (ABA), and methyl jasmonate (MeJA) treatments and significantly upregulated under low temperature, drought, and salt stress; they also exhibited tissue-specific expression patterns. Subcellular localization experiments revealed that *PeJRL04* and *PeJRL13* were localized to the cell membrane, nucleus, and cytoplasm. Three dimensional structure prediction and yeast two-hybrid assays were used to verify that *PeJRL13* exists as a self-interacting homodimer *in vivo*. These findings provide an important reference for understanding the functions of specific moso bamboo JRL genes and for the effective selection of stress-related genes.

## Introduction

Plant lectins are a structurally complex class of sugar-binding proteins that can be divided into several families (Damme et al., 2008). They contain at least one non-catalytic structural domain capable of reversibly binding sugars, and they can trigger a series of downstream biochemical cascades by binding to specific carbohydrates (Peumans and Van Damme, 1995; Sharon, 2007). Lectins play an important role as storage proteins (Barbieri et al., 2004) and participate in biological nitrogen fixation, the promotion of cell division (Jung et al., 2007), endogenous regulation (Xin et al., 2009), and abiotic stress response (Zhang et al., 2000; Ray et al., 2012).

Jacalin-related lectins (JRLs) are a new subfamily of plant lectins that has been recognized in recent years, and they are widely distributed among plant species (Bourne et al., 2004). JRLs have been divided into three categories based on the structural characteristics of their subunits: partial lectins that contain only one jacalin structural domain, chimeric lectins that contain other types of domains, and total lectins that contain multiple jacalin structural domains (Damme et al., 2008). JRLs can also be divided into two classes according to their carbohydrate-binding specificity: galactose-specific JRLs (gJRLs) and mannose-specific JRLs (mJRLs) (Peumans et al., 2001). The gJRLs are mainly localized in storage vesicles, whereas the mJRLs are typically localized in the cytoplasm and nucleus (Peumans et al., 2000). The structural domains of JRL proteins are relatively conserved, but their overall sequence similarity is low (Raval et al., 2004). In addition to having one or more jacalin structural domains, the chimeric JRLs also contain other structural domains at the N- or C-terminus. Common structural domains in monocots include dirigent (20.2%), RX-CC like (7.1%), PKc_like (6.9%), and P-loop_NTPase structural domains (6.8%), and more than 10% of the JRL lectins contain multiple jacalin structural domains (Song et al., 2014). For example, *OsJAC1* and wheat *VER2* have a dirigent structural domain embedded in the N-terminus (Wang and Ma, 2005; Jiang et al., 2006), and it is associated with lignin and lignan formation (Davin et al., 1997; Burlat et al., 2001). Three-dimensional structural analysis of various plant JRL proteins has shown that they exhibit a classical β-prism structure with three sets of β-folded lamellae that are parallel to the axis of the β-prism (van Ooijen et al., 2008). The JRL proteins often form multimers consisting of two to eight monomers (Damme et al., 2008).

Since the first identification of the *JRL* gene in jackfruit (*Artocarpus heterophyllus*) seeds (Bunn-Moreno and Campos-Neto, 1981), *JRLs* have been reported in more than ten plant species. As early as Claes et al. (1990), showed that *Oryza sativa JRL* expression was induced by salt stress (Zhang et al., 2000). Recent research on plant *JRLs* has focused on their functions in stress response (Song et al., 2014). For example, the wheat *TaHfr-1* gene is induced by *Mayetiola destructor* feeding (Subramanyam et al., 2008), and the *O. sativa Salt* gene is induced by drought and hormone treatment and shows high tissue specificity (Jiang et al., 2006). The *Arabidopsis* RTM1 protein is similar to JRL, and its corresponding gene is induced by tobacco etch virus (TEV), inhibiting its long-range transmission (Chisholm et al., 2001). In addition to the stress treatments, the JRL lectin gene can also respond to a variety of plant hormones. For instance, the *TaJRL1* gene in wheat responds to pathogen infestation as well as salicylic acid (SA) and methyl jasmonate (MeJA) treatments (Xiang et al., 2011). Sunflower *HTA1*, sweet potato *Ipomoelin*, and *O. sativa OsJAC1* respond to MeJA treatments (Imanishi et al., 1997; Nakagawa et al., 2000; Jiang et al., 2006), and barley *Lem2* expression is strongly upregulated by SA treatment and downregulated by abscisic acid (ABA) treatment (Abebe et al., 2005). Recent studies have shown that *PeDJ01* (a dirigent-jacalin chimeric gene) is consistently upregulated in moso bamboo leaves under salt and cold stress, suggesting that it may have a role in leaf protection against abiotic stress (Ma et al., 2021).

The economically important non-timber forest species moso bamboo (*Phyllostachys edulis*) originated in Asia and is now cultivated worldwide. With its fast growth rate, high yield, and rapid accumulation of significant biomass during the lignification process, moso bamboo helps to combat global environmental degradation, and forest resource scarcity (Li et al., 2018; Ramakrishnan et al., 2020). However, stressors such as drought and salt (in soil and water) are the main abiotic factors that reduce crop productivity, severely limiting growth and yield (Atkinson and Urwin, 2012; Tadic et al., 2021). The average yield of most major crops is reduced by more than 50% when stress conditions are applied (Wang et al., 2003), and moso bamboo, like other plants, experiences multiple stress conditions during growth and development. These trigger a series of physiological and biochemical changes that ultimately affect the yield and quality of bamboo timber and shoots (Ramakrishnan et al., 2020).

Advances in crop genome sequencing have enabled the identification of JRL genes in species such as *Arabidopsis* (Nagano et al., 2008), *O. sativa* (Jiang et al., 2010), and wheat (Song et al., 2014). However, the possible biological functions of *JRLs* have mainly been analyzed from an evolutionary perspective; less work has been done on their functional characterization, particularly in bamboo. Therefore, in this study, we used bioinformatics approaches to identify JRL family members in the moso bamboo genome and analyzed their evolutionary relationships, gene structures, conserved structural domains, *cis*-acting elements, duplication patterns, and subcellular localization, based on the newly released moso bamboo’s chromosome level reference genome (1,908 Mb, tetraploid) (Zhao et al., 2018). Quantitative real-time PCR (qRT-PCR) was used to analyze the expression patterns of *PeJRL* genes under various hormone treatments and stresses, and the yeast two-hybrid assay using the double-deficient strain AH109 (Trp and Leu) was also performed to verify that the chimeric *PeJRL13* functions as a homodimer. The results of this study provide a reference for future research on stress resistance mechanisms involving *JRL* genes in moso bamboo.

## Materials and Methods

### Identification and Characterization of the *PeJRLs*

Genomic data with the most recent annotations were downloaded from the *P. edulis* genome database^1^. A hidden Markov model file of the conserved JRL structural domain (Pfam01419) was downloaded from Pfam^2^ and used as a seed model to search the bamboo genome database with HMMER v3.2^3^, using a search threshold of *E*-value < 1 × 10^–20^ (Finn et al., 2011). To confirm the search results, we searched the SMART^4^ (Letunic et al., 2012), Pfam (Finn et al., 2016), and InterPro^5^ (Mitchell et al., 2019) databases for the presence of jacalin functional domains, then manually excluded any sequences that lacked the full jacalin functional domain. The resulting *JRL* genes were renamed according to their positions on the bamboo scaffolds.

Subcellular localization predictions were generated with Cell-PLoc 2.0^6^ (Chou and Shen, 2010), and the ExPASy website^7^ (Wilkins et al., 1999) was used to predict the molecular weight (kDa), isoelectric point (PI), and grand average of hydropathicity (GRAVY) of each JRL protein. The SignalP-5.0 online server^8^ was used to predict whether the proteins contained signal peptides.

### Sequence Alignment and Phylogenetic Tree Construction

Whole-genome data for *O. sativa* (*Oryza sativa*) and *Z. mays* (*Zea mays*) were downloaded from the *O. sativa* Genome Annotation Project database^9^ and the Phytozome v.13 database^10^, respectively. *Brachypodium distachyon* genomic data were downloaded from the Ensembl Plants database^11^.

Twenty-nine *O. sativa* JRL proteins, 19 *Z. mays* JRL proteins, and 19 *B. distachyon* JRL proteins were identified by HMMER3 searches of the corresponding local protein databases using the methods described above (Finn et al., 2011). A multiple sequence alignment of all the JRL proteins was produced with MAFFT (v.7.487) (Katoh and Standley, 2013) and used to construct a maximum likelihood (ML) phylogenetic tree with 1000 bootstrap replicates in MEGA X (Kumar et al., 2018).

The core jacalin structural domain sequences were aligned with ClustalX 2.0 (Larkin et al., 2007), and the results were visualized with GeneDoc (v.2.6.002)^12^. Heltuba, a mannose-binding JRL from the Jerusalem artichoke (*Helianthus tuberosus*), was used as the structural reference (Bourne et al., 1999). The carbohydrate-binding site of the moso bamboo jacalin structural domain was predicted with reference to the sugar-binding sites of banana lectin (banlec) and heltuba (Meagher et al., 2005).

### Gene Structures, Motifs, and Conserved Domains

Information on the intron-exon organization of the *PeJRL* genes was extracted from the moso bamboo genome annotation file (GFF3). MEME Suite (v5.3.3)^13^ was used to analyze conserved motifs in the predicted proteins using a search motif value of 8, a minimum width ≥ 10, and a maximum width of 50 (Bailey et al., 2009). TBtools (v.1.0.986) was used to visualize the gene structures and conserved protein motifs (Chen et al., 2020). The NCBI Conserved Structural Domain Database CDD tool^14^ was used to predict conserved structural domains, which were then visualized using IBS1.0 software (Liu et al., 2015).

### Analysis of *cis*-Acting Elements

The genomic sequences 2,000 bp upstream of the transcription start sites (ATG) of the *JRL* genes were extracted from the *P. edulis* genome, and PlantCARE^15^ was used to predict and analyze the *cis*-acting elements in the promoter regions (Magali et al., 2002).

### Gene Distribution, Synteny Analysis, and *K*_a_/*K*_s_ Ratios

Information on the scaffold locations of the moso bamboo JRL genes was obtained from the respective GFF files along with chromosome length information for *O. sativa, Z. mays*, and *B. distachyon*. BLASTP (*E*-value ≤ 10^–5^, other parameters set to defaults) was used to compare the *P. edulis* protein sequences with one another and with those of *O. sativa*, *Z. mays*, and *B. distachyon*. The Multiple Collinearity Scan toolkit (MCScanX) was used to identify gene duplication events and syntenic relationships (Wang et al., 2012). The results were uploaded to the Advanced Circos program^16^ for visualization (Chen et al., 2020).

*K*_a_*K*_s__Calculator2.0 was used to calculate the synonymous substitution rate (*K*_s_), non-synonymous substitution rate (*K*_a_), and *K*_a_/*K*_s_ ratio between homologous *Pe*-*Pe*, *Pe*-*Os*, *Pe*-*Zm*, and *Pe*-*Bd* gene pairs based on coding sequence (CDS) data (Wang et al., 2009). Evolutionary divergence times within the *JRL* gene family were calculated based on the bamboo-specific divergence time formula *T* = *K*_s_/2λ (where λ = 6.5 × 10^–9^) (Peng et al., 2013).

### Gene Ontology Enrichment Analyses and Homology Modeling

We used GOATOOLS^17^ (Klopfenstein et al., 2018) to assign Gene Ontology (GO) annotations to the JRLs and obtain specific biological functions for each JRL protein. Hypergeometric tests were used to test for statistical enrichment of specific GO terms in the JRLs using a false discovery rate (FDR)-corrected *P*-value < 0.05 (Benjamini and Hochberg, 1995). The 3D structure of the JRL protein was predicted using AlphaFold2 (Jumper et al., 2021), and the model structure was visualized and manipulated with PyMOL (The PyMOL Molecular Graphics System, Version 2.4.0).

### Plant Materials and Treatments

Moso bamboo seeds were harvested from Guilin, Guangxi, China, and seedlings were cultured in a greenhouse for 1 month with a 16 h light/8 h dark photoperiod and an average temperature of 22°C. Moso bamboo seedlings with uniform growth was selected and divided into roots, stems, young leaves (unexpanded leaves of the leaf sheath), and mature leaves (fully expanded leaves). Three biological replicates of each tissue type were used for tissue-specific expression analysis. For hormone treatments, the leaves of moso bamboo seedlings were sprayed with 100 μM solutions of ABA, methyl jasmonate (MeJA), or salicylic acid (SA) (Liu et al., 2018) for 0, 3, 6, 12, 24, or 48 h. For control treatments, leaves were sprayed with a water solution and harvested at the same time points. For abiotic stress treatments, seedlings were irrigated with 30% PEG6000 or 200 mM NaCl solution to simulate drought and salt stress, respectively (Chen et al., 2017). For cold treatment, seedlings were exposed to 4°C for 0, 3, or 6 h. Three replicate samples of each treatment and its respective control were harvested, and the leaf tissues were immediately snap-frozen in liquid nitrogen and stored at −80°C.

### RNA Extraction, Reverse Transcription, and Quantitative Reverse-Transcription PCR

Total RNA was isolated from each sample using the FastPure Plant Total RNA Isolation kit (Nanjing Vazyme Biotech Co., Ltd.). First-strand cDNA was synthesized using the Prime-Script RT Reagent Kit (TaKaRa, Dalian, China) according to the manufacturer’s instructions. Specific primers were designed using Beacon Designer 7.0, and all primer sequences are provided in Supplementary Table 1. *PeNTB* was used as the internal reference gene (Fan et al., 2013). The CFX-96 Real-Time system (Bio-Rad, United States) was used to perform qRT-PCR analysis of three technical replicates per sample according to the instructions of TB Premix Ex Taq II (TaKaRa). The reaction program was 95°C for 30 s, followed by 39 cycles of 94°C for 5 s, 60°C for 30 s, and 72°C for 10 s. Relative gene expression was calculated using the 2^–ΔΔ^*^C^*^t^ method (Livak and Schmittgen, 2001) and expressed as mean ± standard deviation (SD). The significance of treatment differences was assessed by one-way ANOVA and visualized using GraphPad Prism 7.

### Sub-Cellular Localization Analysis

Subcellular localization assays were performed following the method of Ma et al. (2021) with the following modifications. Sequence analysis and gene cloning were performed for the *JRL* gene family members *PeJRL04* and *PeJRL13* in order to construct pCAMBIA1300-35S-*PeJRL04*-GFP and pCAMBIA1300-35S-*PeJRL13*-GFP fusion expression vectors. The recombinant plasmids were introduced into *Agrobacterium tumefaciens* GV3101, and the bacteria were resuspended in 20 ml of resuspension buffer (10 mM MES, 10 mM MgCl_2_, and 0.1 mM acetosyringone) to OD_600_ = 0.7. The resuspension was left for 2 h in the dark, and the tobacco was placed under a white fluorescent light for 1 h. The final suspension was injected into the back of the tobacco leaf. After being kept overnight at 22°C in the dark, the leaves were transferred to a 16 h light/8 h dark cycle for 2 days, and the locations of GFP expression were detected using a laser confocal microscope (LSM880, Zeiss, Germany). The pCAMBIA 1300 35S:GFP empty vector was used as the control.

### Yeast Two-Hybrid Assays

The CDSs of *PeJRL04* and *PeJRL13* were amplified by PCR (Supplementary Table 1), and the resulting products were cloned into the pGBKT7 vector and the pGADT7 vector. Yeast two-hybrid assays were performed using the Matchmaker GAL4 Two-Hybrid System (Clontech, United States). The constructs were co-transformed into the yeast strain AH109, and the presence of the target transgene was confirmed by growth on SD/-Leu/-Trp plates. To assess protein interactions, transformed yeast were tested on SD/-Ade/-His/-Trp/-Leu/X-α-Gal (4 mg/mL) medium. The cultures were incubated at 28°C and observed after 3 days. The experiment was performed using three replicates.

## Results

### Identification of *JRL* Genes in Moso Bamboo

Members of the *PeJRL* gene family were identified in a local database of moso bamboo protein sequences using HMMER3 with an *E*-value threshold of 10^–20^. After manually removing redundant genes and genes with incomplete structural domains, we obtained 25 *PeJRL* members and named *PeJRL01*–*PeJRL25* in descending order according to their positions on the genome scaffolds. As shown in Table 1, the CDS lengths of the *PeJRL* genes ranged from 408 (*PeJRL07*) to 3,849 bp (*PeJRL03*), and they encoded proteins ranging from 135 to 1,282 aa. The protein molecular weights ranged from 14.29 kDa (*PeJRL07*) to 146.44 kDa (*PeJRL03*), with theoretical isoelectric points (pIs) between 5.49 (*PeJRL02*) and 9.85 (*PeJRL12*). Most (74.2%) of the JRL proteins had negative GRAVY scores and were therefore hydrophilic. SignalP-5.0 analysis showed that only *PeJRL12* contained a signal peptide, and the remainder of the JRL proteins did not contain. Plant-mPLoc subcellular predictions showed that the moso bamboo JRL proteins were located in the cytoplasm, chloroplast, cell wall and nucleus; nearly half (48%) were located in the cytoplasm.

**TABLE 1:** Detailed information on 25 *JRL* genes from moso bamboo and their encoded proteins.

Gene ID	Gene name	Chromosome location	CDS length (bp)	Size (aa)	MW (kDa)	PI	GRAVY	Signal peptide	Predicted location
PH02Gene41919.t1	*PeJRL01*	S1:131258–137,872	1218	405	43.82	5.72	−0.221	No	C
PH02Gene45488.t1	*PeJRL02*	S1:391224–398,839	936	311	34.24	5.49	−0.086	No	Cm
PH02Gene44052.t1	*PeJRL03*	S1:16856168–16,883,037	3849	1282	146.44	6.32	−0.247	No	C Cy
PH02Gene39026.t1	*PeJRL04*	S3:178076–179,275	459	152	15.85	7.82	−0.214	No	Cw Cy
PH02Gene39024.t2	*PeJRL05*	S3:272862–274,282	582	193	20.24	5.91	−0.171	No	C Cy
PH02Gene43609.t1	*PeJRL06*	S7:39137218–39,140,716	1788	595	65.15	8.62	−0.467	No	Cw
PH02Gene23775.t1	*PeJRL07*	S7:63558624–63,560,470	408	135	14.29	6.05	−0.261	No	C Cy M
PH02Gene23777.t1	*PeJRL08*	S7:63598179–63,600,076	927	308	33.71	9.22	−0.167	No	Cm Cw C Cy M
PH02Gene23778.t1	*PeJRL09*	S7:63606614–63,608,798	1038	345	37.62	9.39	−0.139	No	Cw
PH02Gene08467.t1	*PeJRL10*	S9:53628308–53,632,080	1788	595	65.17	6.84	−0.444	No	Cm Cw Cy N
PH02Gene21121.t1	*PeJRL11*	S9:58690654–58,691,964	738	245	26.01	8.9	−0.2	No	Cm C M
PH02Gene03442.t1	*PeJRL12*	S12:2183398–2,184,761	708	235	25.25	9.85	0.011	Yes	Cm M
PH02Gene03445.t1	*PeJRL13*	S12:2328758–2,330,820	921	306	32.97	5.9	−0.132	No	Cm Cw Cy
PH02Gene25288.t1	*PeJRL14*	S13:34429527–34,433,912	1326	441	49.58	6.02	−0.052	No	C
PH02Gene23335.t2	*PeJRL15*	S13:44327422–44,329,402	495	164	16.84	8.03	0.004	No	C
PH02Gene31836.t1	*PeJRL16*	S14:45892422–45,895,277	846	281	31.01	6.3	−0.454	No	C Cy M
PH02Gene06516.t1	*PeJRL17*	S14:51389578–51,390,436	621	206	21.98	9.23	−0.248	No	Cw Cy M
PH02Gene00698.t1	*PeJRL18*	S16:29562716–29,574,008	1770	589	64.43	8.52	−0.447	No	Cw N
PH02Gene25354.t1	*PeJRL19*	S16:39428929–39,429,633	528	175	19.55	7.81	−0.505	No	Cw
PH02Gene25289.t1	*PeJRL20*	S16:60286083–60,288,845	1620	539	60.19	5.77	−0.069	No	Cm
PH02Gene07107.t1	*PeJRL21*	S22:6702671–6,703,342	450	149	16.35	5.92	−0.168	No	Cy
PH02Gene07832.t1	*PeJRL22*	S22:36027877–36,029,694	918	305	32.41	7	−0.015	No	Cw
PH02Gene30599.t2	*PeJRL23*	S23:67061560–67,063,295	573	190	20.57	6.13	−0.338	No	Cm Cw C Cy M N
PH02Gene30596.t1	*PeJRL24*	S23:67254095–67,258,156	678	225	25.38	5.73	−0.591	No	Cw C
PH02Gene48979.t1	*PeJRL25*	S23:75129747–75,132,374	426	141	14.55	6.38	0.11	No	Cw Cy

*MW, molecular weight. pI, isoelectric point. GRAVY, grand average of hydropathicity score. N, nucleus. C, chloroplast. M, mitochondrion. Cm, cell membrane. Cw, cell wall. Cy, cytoplasm.*

### Phylogenetic Analysis of the *JRL* Family

**FIGURE 1:**
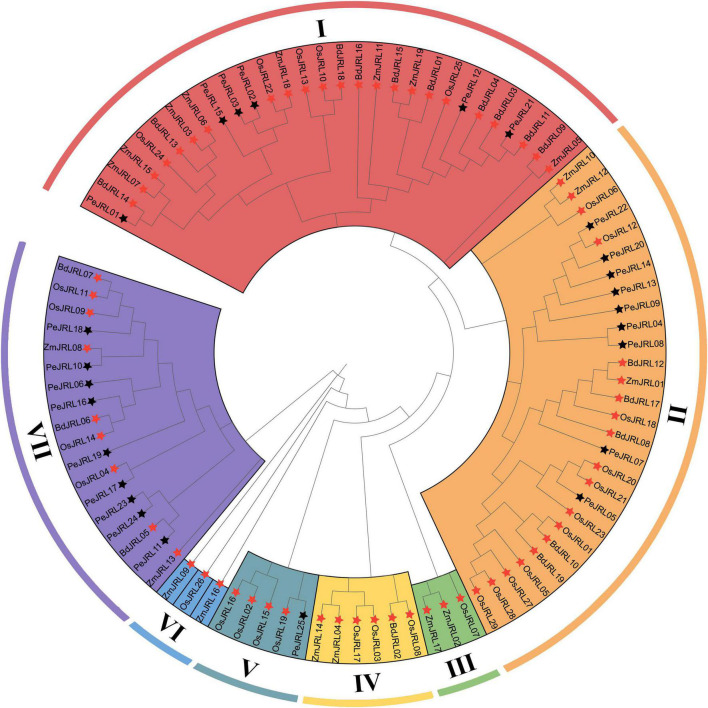
Phylogenetic analysis of full-length JRL protein sequences from *Phyllostachys edulis* (Pe, bamboo), *Oryza sativa* (Os, rice), *Zea mays* (Zm, maize), and *Brachypodium distachyon* (Bd). MAFFT was used to build a multiple sequence alignment, and MEGAX was used to construct a maximum likelihood (ML) phylogenetic tree with 1,000 bootstrap replicates. Black stars indicate moso bamboo sequences, and red stars indicate those of other species.

To clarify the evolutionary relationships among PeJRL proteins from different grasses, we constructed a maximum likelihood (ML) phylogenetic tree from 92 JRL protein sequences: 25 from *P. edulis*, 29 from *O. sativa*, 19 from *Z. mays*, and 19 from *B. distachyon*. With reference to a previously reported classification (Song et al., 2014), we classified the JRL proteins of the different species into seven subgroups (I–VII) (Figure 1). Subgroup I was the largest, with 29 members, followed by subgroup II (28), whereas subgroups III and VI had the fewest members (3). The largest number of *O. sativa* JRLs occurred in subgroup II (11). The 25 PeJRLs were distributed only in subgroups I (6 members), II (9), V (1), and VII (9). Three pairs of orthologs were identified between moso bamboo and *O. sativa* (*PeJRL17*-*OsJRL04*, *PeJRL25*-*OsJRL19*, and *PeJRL22*-*OsJRL12*), three between bamboo and *B. distachyon* (*PeJRL01*-*BdJRL14*, *PeJRL21*-*BdJRL11*, and *PeJRL11*-*BdJRL05*), and one between bamboo and *Z. mays* (*PeJRL10*-*ZmJRL08*).

### Homology Modeling and Multiple Sequence Alignment of Jacalin Domains

**FIGURE 2:**
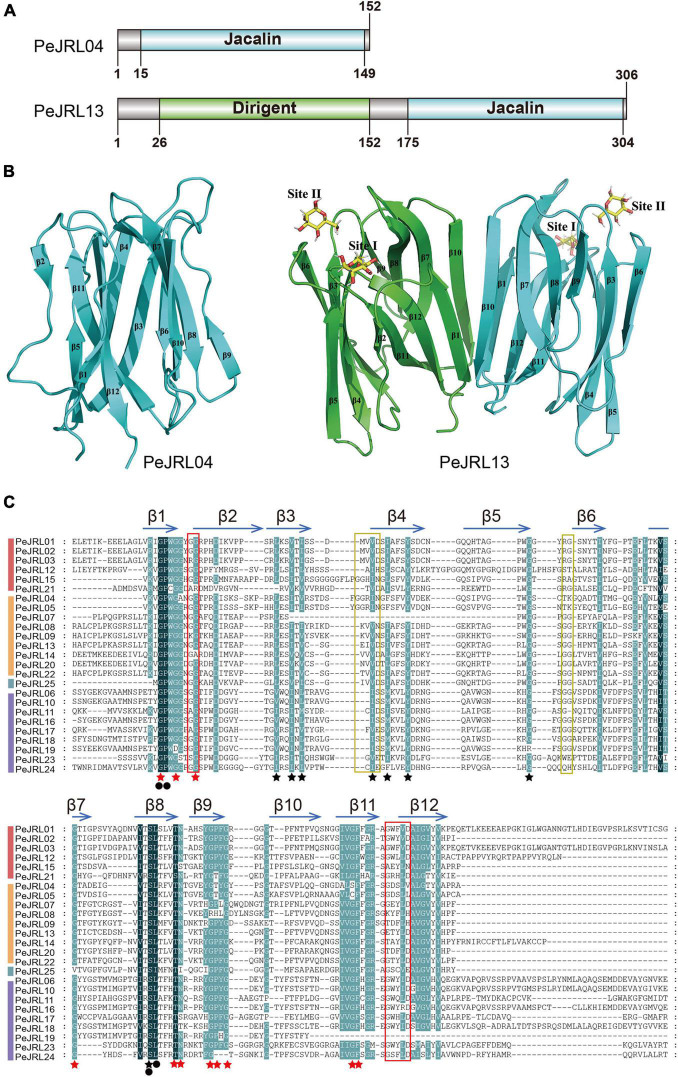
Different views of the structural domains of the moso bamboo JRLs. **(A)** Domain diagram of two PeJRL proteins. **(B)** Ribbon diagrams of the predicted three-dimensional structures of the jacalin monomer *PeJRL04* and the dimer *PeJRL13*. Sites I and site II are the two sugar-binding sites. **(C)** Multiple sequence alignment of the jacalin structural domains of the *PeJRLs*. The background colors emphasize the level of similarity: dark indicates 100%, and light indicates ≥80%. Regions corresponding to β-strands are indicated by horizontal arrows above the sequences, and red stars indicate key residues in heltuba used to package the three Greek key motifs, identified using heltuba as a reference (Bourne et al., 1999). Black stars indicate conserved residues that were not reported by Bourne et al. (1999). Invariant residues are indicated by filled black circles. The first and second sugar-binding sites in heltuba are enclosed by red and yellow rectangles, respectively.

The homology modeling results are shown in Figure 2B. *PeJRL04* consists of a jacalin monomer with a typical β-prismatic structure that includes three sets of β-folded lamellar structures, each one parallel to the axis of the prism. Within the prismatic structure, the C-terminal portion located in the α-strand forms 11-fold, and the N-terminal portion located in the β-strand creates the 12th β-fold. By contrast, *PeJRL13* is predicted to be a dimer formed by jacalin (blue) and dirigent (green) monomers. Both *PeJRL04* and *PeJRL13* contain two glycan binding sites on each monomer, Site I and Site II. The homology modeling results were consistent with the predicted structural domain analysis (Figure 2A), indicating that members of the *PeJRL* family can form diverse multimeric structures.

To identify conserved features of the jacalin core domain in moso bamboo, we constructed a multiple sequence alignment of the jacalin structural domains of the PeJRL proteins. We then compared them to the well-established structures of the mannose-binding JRL domains from heltuba (Bourne et al., 1999) and banana lectin (banlec) (Meagher et al., 2005). Forty-four highly conserved residues with a similarity of at least 80% were identified. Of the 12 key residues necessary for the integrity of the β-prism fold in heltuba, 11 are conserved in the *PeJRLs*; the exception is the phenylalanine residue on β10, which is missing. Gly and Pro residues in β1 and Ser and Leu residues in β8 are invariant in all *PeJRL* jacalin structural domains, suggesting that they are essential for the protein’s biological function. The Gly residues involved in the mannose binding of heltuba and banlec are present between the β1 and β2 loops of all PeJRL proteins except *PeJRL21* (subgroup I) and *PeJRL14* (subgroup II). However, the complete ligand-binding loop (G-X3-D motif) described for banlec and located between the β11 and β12 chains was present in only 15 of the PeJRL proteins (60%). The GG loop motif of the second carbohydrate binding site was also present in the β5–β6 chains of the jacalin domain in some PeJRL proteins (56%) (Figure 2C).

### Gene Structures, Motifs, and Domain Compositions

Gene structure analysis revealed (Supplementary Figure 1A) that all JRL genes in *P. edulis* are less than 1 kb in length, with the exceptions of *PeJRL03* (2.7 kb) and *PeJRL18* (1.8 kb). Most genes have 3–6 introns, although *PeJRL03* has 9 introns. We used MEME tools to identify eight conserved motifs in the PeJRL proteins (Supplementary Figure 1B). All members of the JRL family contain motif 2, except *PeJRL25*, which is the sole bamboo member of subgroup V. Motifs 1, 2, 5, and 7 together make up the jacalin structural domain (Supplementary Figure 1C). Interestingly, motif 3 is specific to members of subgroup VII, and motif 4 is found only in subgroups I, II, and V.

Using a previous classification scheme based on the number of jacalin domains and the presence or absence of other structural domains (Song et al., 2014), we identified 19 type I JRL proteins, 6 type II JRL proteins, and no type III or IV JRL proteins in moso bamboo (Supplementary Figure 2). The genes that contained only one jacalin domain had the highest percentage of type I proteins (74%). Notably, in addition to the jacalin domain, some PeJRL proteins also appear to contain one or more structural domains of other types. For example, PeJRL03 also contains RX-CC_like, NB-ARC, and PKc_like structural domains, and PeJRL08, 09, 13, and 22 all contain jacalin-dirigent chimeric structural domains.

### Analysis of *cis*-Acting Elements in *PeJRL* Promoters

**FIGURE 3:**
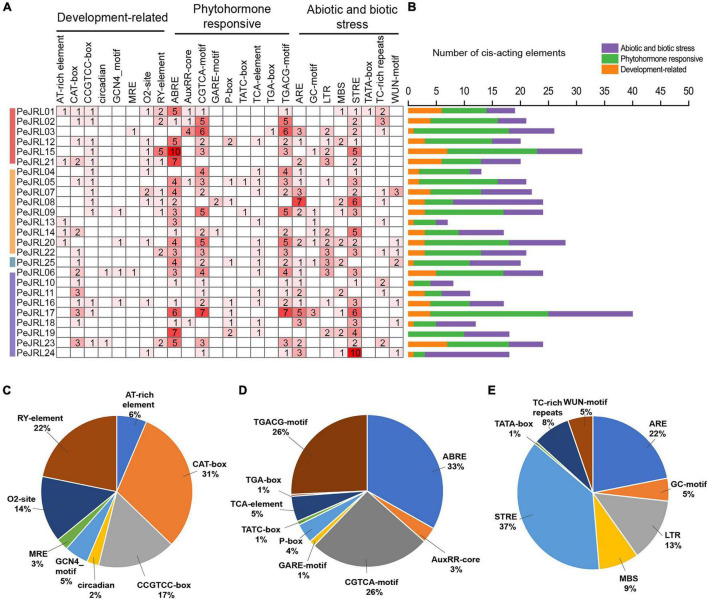
*Cis*-acting elements in the *PeJRL* promoters of moso bamboo. **(A)** The intensity of the red color and the numbers in the cells indicate the numbers of different *cis*-acting elements in each *PeJRL*. **(B)** The colored histograms indicate the number of different *cis*-acting elements in three categories. **(C–E)** The proportions of different *cis*-elements in each category: **(C)** development-related, **(D)** phytohormone responsive, and **(E)** abiotic and biotic stress responsive.

*Cis*-acting elements are located near genes and participate in the regulation of gene expression. To analyze the potential response mechanisms of the *PeJRL* genes, we used PlantCARE to analyze the promoter sequences 2,000 bp upstream of the *JRL* start codons. We identified 506 *cis*-acting elements in the 25 *PeJRL* promoters; they could be divided into three categories: development-related, phytohormone responsive, and abiotic and biotic stress responsive (Figure 3A). With the exception of *PeJRL19*, each *PeJRL* promoter contained *cis*-elements from all three categories (Figure 3B). In the development-related category (78/506), the CAT-box element related to meristem-specific expression was most abundant (31%), followed by the RY-element related to seed formation (22%). *Cis*-elements related to the regulation of growth metabolism such as the CCGTCC-box (17%), O2-site (14%), and AT-rich element (6%) were also detected in JRL promoters (Figure 3C). A large number of *cis*-elements belonged to the phytohormone responsive category (241/506). The abscisic acid (ABA) response-related ABRE (33%) element was the most abundant (>80) and was present in almost all *PeJRL* promoters. The CGTCA-motif (26%) and TGACG-motif (26%) related to the MeJA response and the TCA-element (5%) associated with the salicylic acid (SA) response were also present, as were the growth hormone-related TGA-box (4%) and AuxRR-core element (3%), the gibberellin-related GARE-motif (1%), the P-box (1%), and the TATC-box (1%) (Figure 3D). We also detected a large number of abiotic and biotic stress responsive *cis*-elements, including elements associated with stress response (STRE, 37%), antioxidant response (ARE, 22%), low temperature response (LTR, 13%), and drought induction (MBS, 9%) (Figure 3E). These results suggest that *PeJRL* genes may be extensively involved in abiotic stress response and that their expression may be induced by hormones.

### Chromosome Locations and Duplications of the *PeJRL* Genes

**FIGURE 4:**
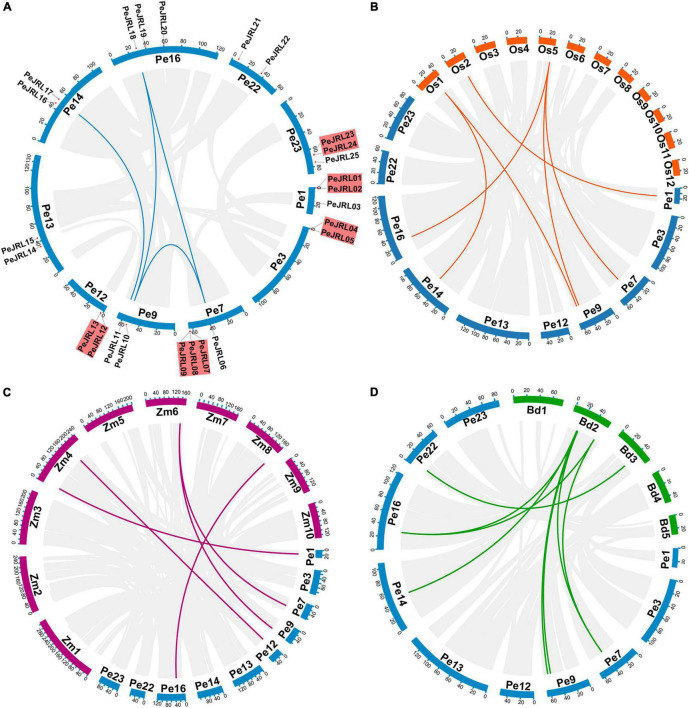
Synteny analysis of the *PeJRL* genes. **(A)** Chromosome distribution and inter-chromosomal relationships of the *PeJRL* genes. Tandem duplicated genes are set off by a red background. Scale bars represent the number of DNA bases in Mb. **(B–D)** Synteny analysis of *PeJRL* genes in *P. eduli*s and three other model plants (*O. sativa*, *Z. mays*, and *B. distachyon*). Gray lines represent aligned blocks between the paired genomes, and red lines indicate syntenic JRL gene pairs. Pe, *P. eduli*s. Os, *O. sativa*. Zm, *Z. mays*. Bd, *B. distachyon*.

The 25 *PeJRLs* were unevenly distributed across the 10 genomic scaffolds of *P. edulis* (Figure 4A). The largest number of genes was found on scaffold 7 (4), followed by scaffolds 1 (3), 16 (3), and 23 (3); all other scaffolds contained two genes.

There are two main types of gene duplication: tandem duplication and segmental duplication (Cannon et al., 2004). In the former, duplicates are typically found in chromosomal recombination regions and appear as gene clusters (Ramamoorthy et al., 2008); in the latter, gene duplicates are more distantly located (Yu et al., 2005). The duplication of *PeJRLs* was analyzed using MCScanX, and the majority of *PeJRL* genes (64%) were located in duplicated regions. There were five tandem duplicated regions on scaffolds 1, 3, 7, 12, and 23 and four pairs of segmental duplicates on scaffolds 7, 9, 14, and 16 (Figure 4A). These results suggest that tandem duplication (44%) and segmental duplication (20%) have been important drivers of *PeJRL* gene family amplification. Genome synteny analysis of moso bamboo and three graminaceous model plants revealed that six, five, and eight moso bamboo *JRL* genes were homologous to *JRL* genes of *O. sativa*, *Z. mays*, and *B. distachyon*. Interestingly, some JRL genes on *O. sativa* chromosomes 1 and 5 corresponded to two or three homologs on different scaffolds of moso bamboo. This phenomenon was also found for *Z. mays* and *B. distachyon* (Figures 4B–D).

### Evolutionary Patterns of the *JRL* Genes

**FIGURE 5:**
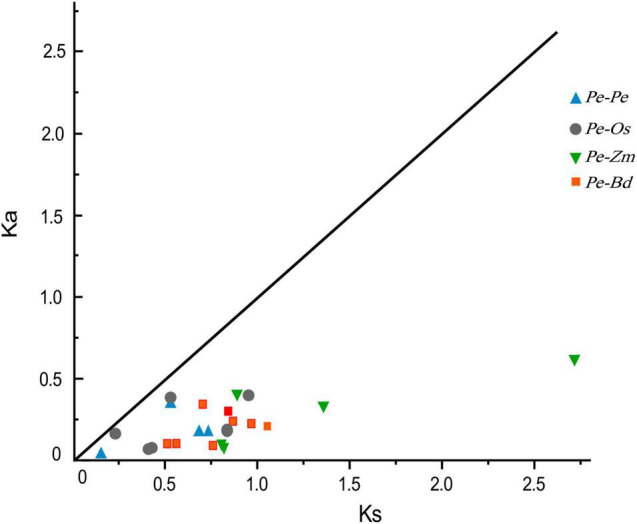
Distribution of *K*_a_ and *K*_s_ values for paralogous (*Pe*-*Pe*) and orthologous (*Pe*-*Os*, *Pe*-*Zm*, and *Pe*-*Bd*) gene pairs. The black line indicates the slope of *K*_a_/*K*_s_ = 1.

Four *Pe*-*Pe*, six *Pe*-*Os*, five *Pe*-*Zm*, and eight *Pe*-*Bd* putative homologous gene pairs were identified by synteny analysis. To investigate the evolution and divergence patterns of the JRLs, *K*_s_ values and *K*_a_/*K*_s_ ratios were calculated for all homologous gene pairs (Supplementary Table 2). The *K*_s_ values for *PeJRL* homologous gene pairs were 0.151–0.737, suggesting that a large-scale gene duplication event may have occurred as early as 11–55 million years ago (MYA). The mean *K*_*s*_ values for homologous JRL gene pairs between species were 0.564 (*Pe*-*Os*), 1.317 (*Pe*-*Zm*), and 0.784 (*Pe*-*Bd*). Notably, previous studies have confirmed that the genomes of moso bamboo and *Z. mays* diverged about 7–12 MYA (Peng et al., 2013), significantly later than the latest divergence of JRL homologs in both species (62 MYA). We therefore concluded that the *JRL* genes had already undergone most of their genetic evolution before the separation of the two species. In addition, *K*_a_/*K*_s_ values for all homologous gene pairs were less than 1.0 (Figure 5), implying that these genes have undergone purifying selection to eliminate deleterious mutations.

### Gene Ontology Enrichment Analysis of the PeJRL Proteins

**FIGURE 6:**
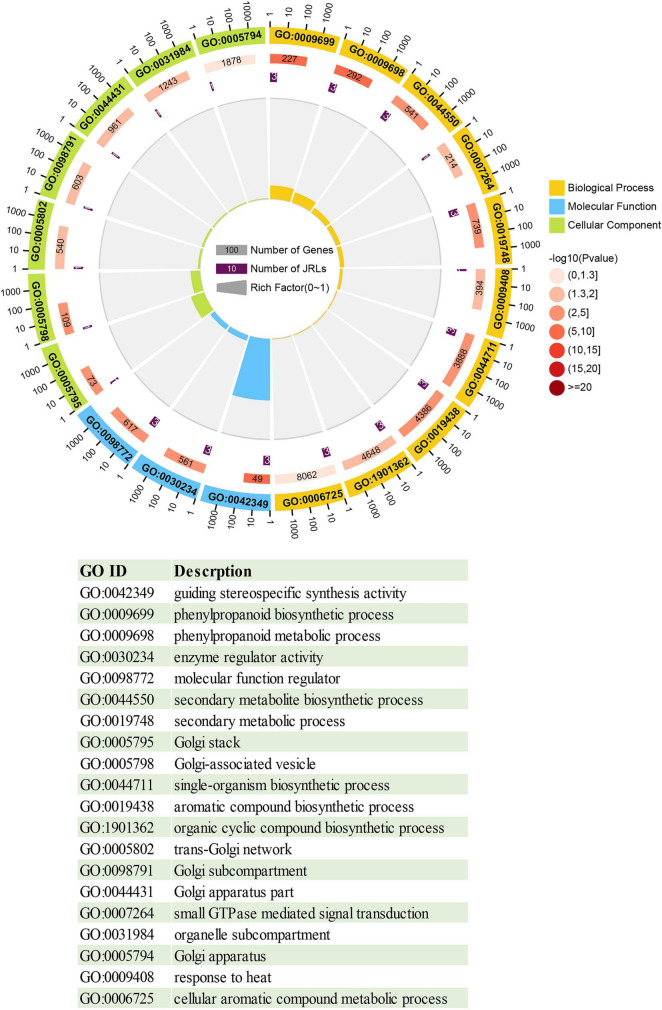
Circle diagram of the top 20 enriched GO terms. From outside to inside, the first ring gives the top 20 significantly enriched GO term IDs, with different colors representing different classifications. The second ring indicates the number of background genes with that classification and the enrichment significance *P*-value of that GO term. The third lap indicates the number of *PeJRL* genes annotated with that term. The fourth ring indicates the enrichment factor for each GO term. Functional descriptions of the GO terms are given below.

To investigate the function of the JRL proteins, we performed GO annotation and enrichment analysis of the PeJRLs relative to the complete GO database. The top 20 enriched GO terms included 10 biological process, 7 cellular component, and 3 molecular function terms (Figure 6 and Supplementary Table 3). The terms ‘guiding stereospecific synthetic activity’ (GO:0042349), ‘phenylpropanoid biosynthetic process’ (GO:0009699), ‘phenylpropanoid metabolic process’ (GO:0009698), and ‘enzyme regulator activity’ (GO:0030234) were associated with the greatest number of proteins (three in each case). ‘Guiding stereospecific synthesis activity’ had the highest enrichment factor (0.061).

### Tissue-Specific Expression Patterns of the *PeJRL* Genes

**FIGURE 7:**
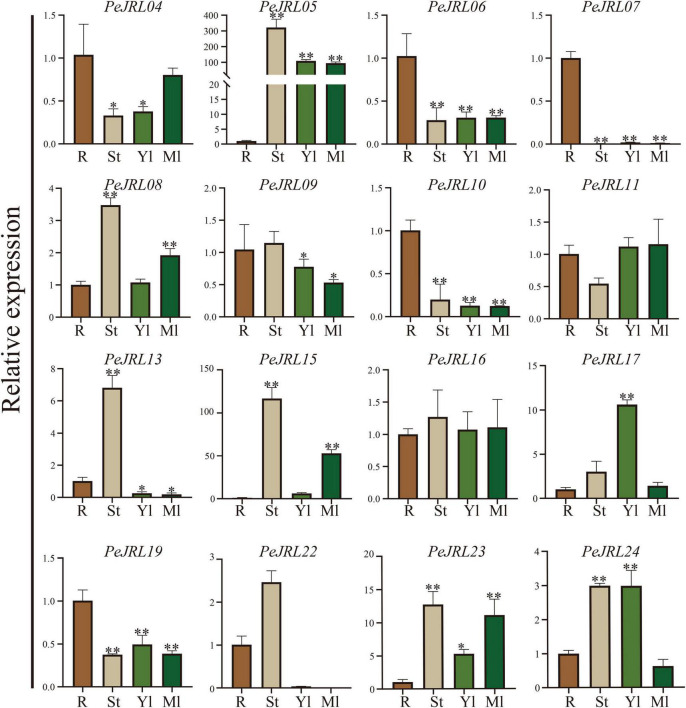
Tissue-specific expression patterns of the JRL genes in moso bamboo. The expression level of each sample in roots was set to 1. qRT-PCR was performed using three biological and three technical replicates of each tissue type. Asterisks indicate statistically significant differences between roots and other tissues (*P ≤ 0.05, ^**^P ≤ 0.001).

We performed qRT-PCR analyses to examine the expression of 12 *PeJRL* genes from three subfamilies in different tissues of January-grown moso bamboo: roots (R), stems (St), young leaves (Yl), and mature leaves (Ml). Although most PeJRLs were expressed in all tissues examined, there were significant differences in their tissue-specific expression patterns. For example, *PeJRL05* expression was about 300-fold higher in stems and about 100-fold higher in leaves than in roots. Likewise, *PeJRL15* expression was more than 100-fold higher in stems than in roots. By contrast, the expression of *PeJRL06*, *07*, *10*, and *19* was much higher in roots than in other tissues, suggesting that these may be primarily root-specific genes (Figure 7).

### Expression Patterns of the *PeJRLs* in Response to Methyl Jasmonate, Salicylic Acid, and Abscisic Acid Treatments

Salicylic acid, ABA, and MeJA were sprayed on 1-month-old leaves of bamboo seedlings, and the expression patterns of 15 *PeJRL* genes were analyzed by qRT-PCR at different time points after treatment application.

**FIGURE 8:**
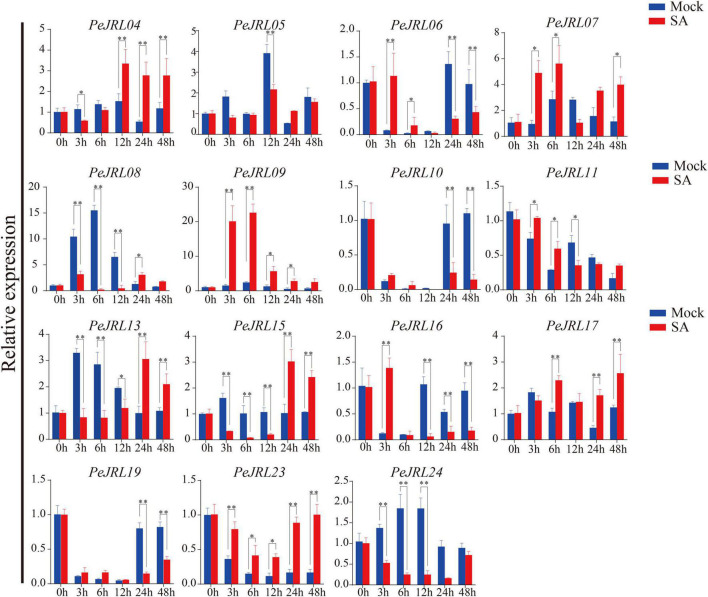
Expression patterns of *PeJRL* genes after SA treatment. Sampling occurred 0, 3, 6, 12, 24, and 48 h after treatment, and the relative expression level of the sample at 0 h was set to 1. The qRT-PCR results were obtained from three biological and three technical replicates. Asterisks indicate statistically significant differences between treated and control samples (*P ≤ 0.05, ^**^P ≤ 0.001).

The expression of three genes (*PeJRL07*, *17*, and *23*) was upregulated compared to the controls at several time points following SA treatment (Figure 8). *PeJRL07* showed a five-fold increase in expression within 3 h compared to the controls. Multiple *JRL* genes showed a pattern of induction followed by repression after SA treatment; these included *PeJRL06*, *11*, and *16*. *PeJRL04*, *15*, and *17* responded later (after 24 h) to SA treatment. By contrast, *PeJRL24* expression was significantly downregulated at all time periods after SA treatment.

**FIGURE 9:**
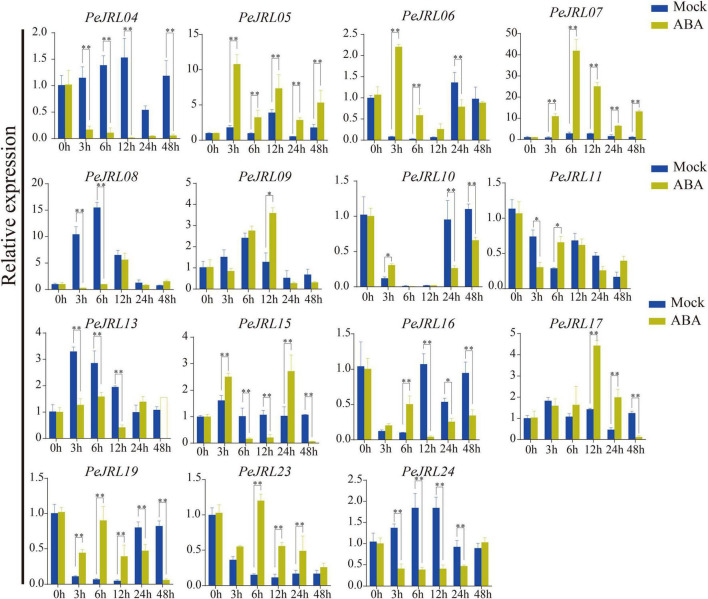
Expression patterns of *PeJRL* genes after ABA treatment. Sampling occurred 0, 3, 6, 12, 24, and 48 h after treatment, and the relative expression level of the sample at 0 h was set to 1. The qRT-PCR results were obtained from three biological and three technical replicates. Asterisks indicate statistically significant differences between the treated and control samples (*P ≤ 0.05, ^**^P ≤ 0.001).

Seven (58%) *PeJRL* genes showed a significant increase in expression soon after ABA treatment (3–6 h) (Figure 9). In particular, *PeJRL05* expression was upregulated more than 10-fold 3 h after spraying, *PeJRL07* expression was upregulated 40-fold 6 h after spraying, and *PeJRL17* expression was upregulated 4-fold after ABA treatment. Two of the genes whose expression was downregulated at almost all time points were *PeJRL04* and *PeJRL24*. *PeJRL06*, *10*, *15*, and *16* showed a pattern of induction followed by inhibition.

**FIGURE 10:**
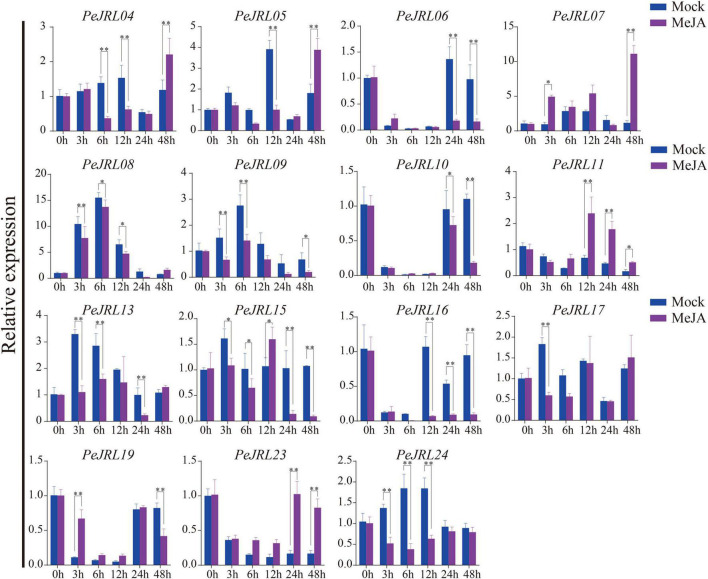
Expression patterns of the *PeJRL* genes after MeJA treatment. Sampling occurred 0, 3, 6, 12, 24, and 48 h after treatment, and the relative expression level of the sample at 0 h was set to 1. The qRT-PCR results were obtained from three biological and three technical replicates. Asterisks indicate statistically significant differences between the treated and control samples (*P ≤ 0.05, ^**^P ≤ 0.001).

Four *JRL* genes were upregulated after MeJA treatment: *PeJRL07*, *11*, *19*, and *23* (Figure 10). Some genes were significantly upregulated 12–48 h after treatment: *PeJRL11* was upregulated more than two-fold 12 h after treatment, and *PeJRL23* was upregulated more than twofold 24 h after treatment. The expression of other genes was downregulated two-fold soon after MeJA application; these included *PeJRL17* and *PeJRL24.* Interestingly, *JRL* members of subgroup VII all showed an increase in expression early in SA treatment (6 h), except for *PeJRL17* and *PeJRL24*, which were slightly downregulated. Similar results were observed in response to ABA and MeJA treatment. Overall, these data are consistent with the predictions of the *cis*-acting element analysis of the *PeJRL* promoters.

### Expression Patterns of the *PeJRLs* Under Different Abiotic Stresses

**FIGURE 11:**
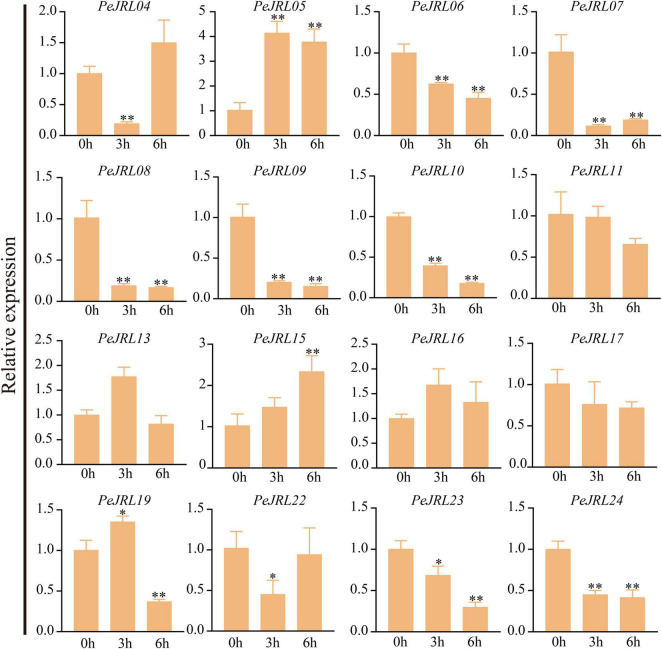
Expression patterns of *PeJRL* genes under low temperature conditions. Sampling occurred 0, 3, and 6 h after treatment, and the relative expression level of the sample at 0 h was set to 1. The qRT-PCR results were obtained from three biological and three technical replicates. Asterisks indicate statistically significant differences between the stressed samples and the 0 h controls (*P ≤ 0.05, ^**^P ≤ 0.001).

Previous studies have shown that *JRL* genes are involved in abiotic stress responses in different plants. To further investigate the effects of various stresses on transcript levels of the *PeJRLs*, we examined their expression patterns at early stages (3–6 h) of low temperature, drought, and salt stress by RT-PCR using 0 h untreated bamboo seedlings as the controls. Seven out of 16 *JRL* genes were significantly repressed compared with the controls at two time periods after low temperature treatment: *PeJRL06*, *07*, *08*, *09*, *10*, *23*, and *24* (Figure 11). Three genes were upregulated: *PeJRL05*, *15*, and *16*. The expression of *PeJRL19* was induced at 3 h and repressed at 6 h.

**FIGURE 12:**
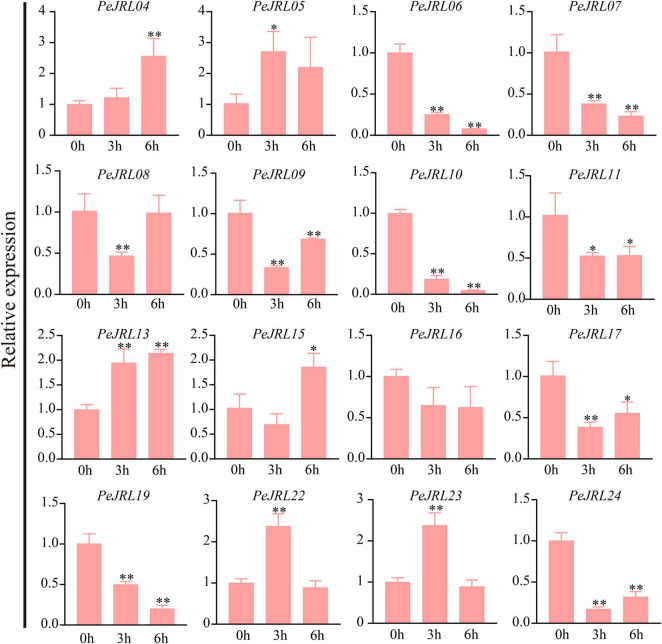
Expression patterns of the *PeJRL* genes under drought conditions. Sampling occurred 0, 3, and 6 h after treatment, and the relative expression level of the sample at 0 h was set to 1. The qRT-PCR results were obtained from three biological and three technical replicates. Asterisks indicate statistically significant differences between the stressed samples and the 0 h controls (*P ≤ 0.05, ^**^P ≤ 0.001).

Under drought stress, the expression of *PeJRL04*, *05*, *13*, *15*, *22*, and *23* increased, and the expression of *PeJRL06*, *07*, *10*, *11*, *17*, *19*, and *24* decreased at all time periods after treatment (Figure 12).

**FIGURE 13:**
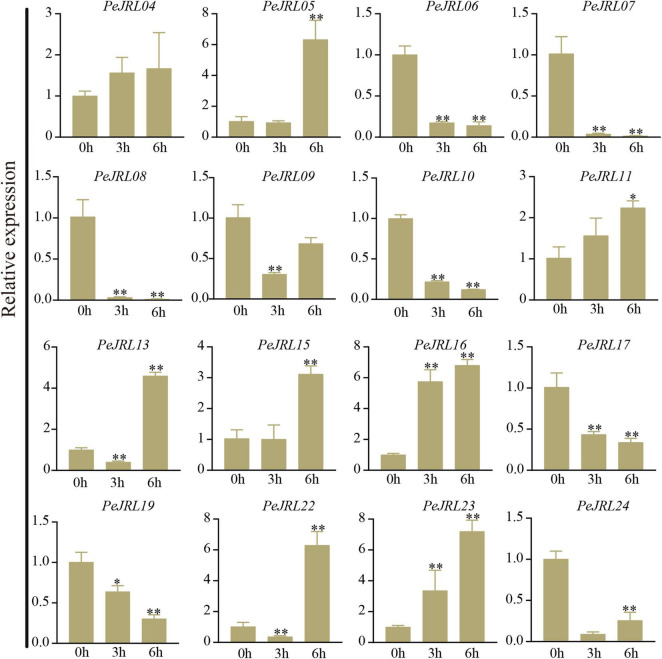
Expression patterns of *PeJRL* genes under salt stress conditions. Sampling occurred 0, 3, and 6 h after treatment, and the relative expression level of the sample at 0 h was set to 1. The qRT-PCR results were obtained from three biological and three technical replicates. Asterisks indicate statistically significant differences between the stressed samples and the 0 h controls (*P ≤ 0.05, ^**^P ≤ 0.001).

Under high salt treatment, about half of the JRL genes were upregulated; these included *PeJRL05*, *11*, *15*, *16*, and *23*. *PeJRL16* was strongly induced by high salt, and its expression was sixfold higher after treatment than in the control (Figure 13).

The subgroup II members *PeJRL04*, *05*, and *13* were upregulated under low temperature, drought, and salt stress, whereas the other three subfamily II members (*PeJRL07*, *08*, and *09*) were consistently downregulated under stress compared with the controls. The expression patterns of *JRLs* from the same subfamily thus differed under different stress treatments, with some showing upregulation and some showing repression.

### Homodimer Detection and Subcellular Localization Analysis of *PeJRL04* and *PeJRL13*

**FIGURE 14:**
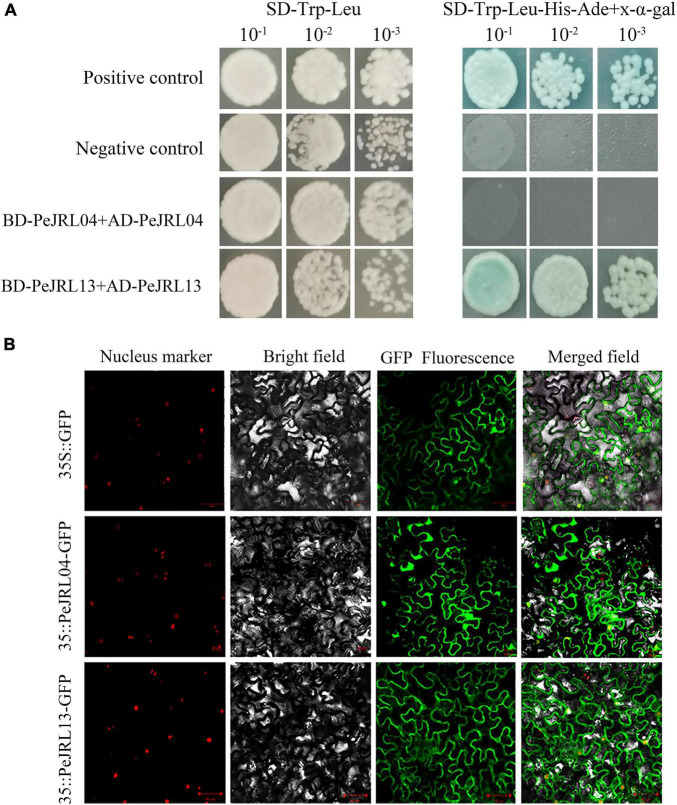
Y2H and subcellular localization assays of *PeJRL04* and *PeJRL13* proteins. **(A)** Y2H assay for homodimer formation in *PeJRL04* and *PeJRL13*. Coding sequences of the *PeJRL* genes were cloned into pGADT7 (AD) and pGBKT7 (BK) vectors to assess potential *PeJRL04–PeJRL04* and *PeJRL13–PeJRL13* interactions. ‘–1’ indicates a 1:10 (100 μl bacteria + 900 μl water) dilution of the resuspension solution transferred with the recombinant plasmid, ‘–2’ indicates a 1:100 dilution, and ‘–3’ represents a 1:1,000 dilution. **(B)** Subcellular localization of 35S:PeJRL04-GFP and 35S:PeJRL13-GFP fusion proteins in tobacco epidermal cells. The 35S:GFP pCambia1300 empty vector was used as the control.

Jacalin-related lectins proteins (Supplementary Table 4) often form multimers with 2–8 monomers. We investigated multimer formation in *PeJRL04* (GenBank MW650828), which contains only one jacalin domain, and *PeJRL13* (GenBank MW650829), a member of the DJRL family that contains both jacalin and dirigent domains. The full length CDSs were ligated into pGADT7 and pGBKT7 vectors by seamless cloning and transformed into the AH109 yeast strain. On the SD/-Trp-Leu plates, the positive control, negative control, and experimental group all grew normally, indicating successful transformation of the recombinant plasmid (Figure 14A). On the SD/-Trp-Leu-His-Ade plates containing *x*-α-gal, both the positive control and *PeJRL13* grew normally, indicating that *PeJRL13* could interact with itself to form a homodimer, whereas *PeJRL04* could not.

To determine the locations of PeJRL protein expression in cells, we fused *PeJRL04* and *PeJRL13* CDS sequences with GFP driven by the CaMV35S promoter and expressed the fusion construct in tobacco epidermal cells. The 35S:GFP pCambia1300 empty vector served as the control. We observed that *PeJRL13* was distributed in the cell membrane, nucleus, and cytoplasm and was therefore a nucleoplasmic protein. *PeJRL04* was distributed mainly in the nucleus and cell membrane, and a small amount was also detected in the nucleoplasm (Figure 14B).

## Discussion

### Genome-Wide Identification and Phylogenetic Analysis of the *PeJRL* Genes

Jacalin-related lectins are widespread in plants and are found in many species, including *Arabidopsis*, *O. sativa*, wheat, *Z. mays*, sorghum, and *B. distachyon* (Nagano et al., 2008; Jiang et al., 2010; Song et al., 2014). Previous studies have demonstrated that the number of *JRL* genes ranges from 46 to 123 in cruciferous plants and from 20 to 41 in graminaceous plants (Han et al., 2019). In this study, we identified 25 *JRL* genes in moso bamboo based on specific criteria. *JRL* genes that encoded a larger number of jacalin structural domains had a larger number of introns, and presumably the number of introns increased with increasing domain number. We also identified 29 *JRLs* in *O. sativa*, 19 in *Z. mays*, and 19 in *B. distachyon* using the same criteria. Gramineae diverged 50–70 MYA (Kellogg, 2001). In general, the graminoids have similar numbers of *JRL* genes, and these genes may have expanded at a similar rate. However, some previous reports have suggested that *P. edulis* contains two duplicated genes for each *O. sativa* gene (Peng et al., 2013). This phenomenon has been reported in many *P. edulis* gene families, such as WRKY and E2F/DP (Li et al., 2017, 2021). There are many more WRKYs and E2F/DPs in *P. edulis* than in *O. sativa*. Interestingly, in the current study, there were far fewer JRL members in moso bamboo than in *O. sativa*, presumably owing to contraction and loss in the JRL gene family of moso bamboo or expansion in *O. sativa*.

Phylogenetic relationships demonstrated that the 92 JRLs from the four monocot species could be divided into seven subgroups (I–VII), as reported in previous studies (Song et al., 2014). Interestingly, one species often occurs disproportionately in specific subgroups, indicating that species-specific expansions have occurred in some JRL lineages (Figure 1). The moso bamboo and *B. distachyon* JRL proteins are often clustered together, suggesting that the moso bamboo and *B. distachyon* JRL proteins are more closely related.

Multiple sequence alignments of the PeJRL proteins revealed low amino acid sequence similarity outside of the jacalin domain. Structurally resolved jacalin structural domains all have the same fold (Damme et al., 2008). In the present study, the jacalin structural domain of the *PeJRL* proteins was compared with that of heltuba, and 11 of the 12 heltuba residues associated with the β-prismatic fold structure were conserved in the *PeJRLs*, with the exception of a missing phenylalanine residue on β10. We hypothesized that plant *JRL* genes have evolved considerably, with the exception of the conserved nucleotides that encode key amino acid sites in the jacalin domain; other protein regions have changed markedly, possibly reflecting the different growth environments of plants and the widely varying environmental stresses they face in nature. We also identified a number of new conserved residues (Figure 2C) whose functions will require further investigation. Similar phenomena have been reported in other graminaceous plants (Meagher et al., 2005).

Under abiotic stress, transcription factors bind to the *cis*-acting elements of stress-responsive gene promoters to specifically initiate transcriptional expression of the corresponding genes. Numerous studies have shown that JRL proteins are involved in hormone signaling and show stress- and tissue-specific expression. For example, studies in *O. sativa* have found that JRL proteins are expressed in response to drought and hormone treatment and have highly tissue-specific expression (Bunn-Moreno and Campos-Neto, 1981). Here, hormone response–related elements, such as those associated with ABA, SA, and MeJA, made up nearly 50% of the *cis*-acting elements in the *PeJRL* promoters (Figure 3). A large number of elements related to abiotic stress and tissue-specific expression were also detected, suggesting that *PeJRL* may participate in the stress responses of moso bamboo, particularly in response to hormone treatment.

### Evolutionary Features of the PeJRL Family

During evolution, gene families expand primarily through segmental and tandem duplication (Cannon et al., 2004; Zhu et al., 2014). Gene family members may be closely spaced on the same chromosome after tandem duplication, forming a gene cluster, or scattered at different locations on one or more chromosomes by segmental duplication (Ohno, 1971; Semple and Wolfe, 1999). Intraspecific syntenic relationships show that approximately 64% of the *PeJRL* genes are located in duplicated regions, including five instances of tandem duplication and four instances of segmental duplication, suggesting that these two types of duplication are the main drivers of expansion of the *PeJRL* gene family. This is consistent with the findings of a previous study on the evolution of large JRL families such as that of *O. sativa*, which hypothesized that JRL genes may have evolved from the ancient jacalin domain through tandem and segmental duplications (Xiang et al., 2011). Here, collinearity analysis with other plant genomes revealed five syntenic JRL gene pairs between *P. edulis* and *Z. mays*, seven between *P. edulis* and *O. sativa*, and eight between *P. edulis* and *B. distachyon* (Figure 4). These results suggests that the *JRL* genes of *P. edulis* are more closely related to those of *B. distachyon* than to those of *O. sativa* and *Z. mays*. Notably, this result is consistent with a chloroplast genome sequence analysis that suggested *B. distachyon* was most closely related to bamboo (Peng et al., 2013).

### Tissue-Specific and Abiotic Stress Responsive Expression

The tissue specificity of *JRL* expression has been demonstrated in some plants, and the results of *cis*-acting element analysis suggested that *PeJRLs* were involved in abiotic stress responses. We therefore randomly selected some *PeJRLs* and studied their tissue-specific, hormone-regulated, and abiotic stress–induced expression patterns to explore the stress tolerance mechanisms of the moso bamboo *JRL* family.

The *PeJRLs* showed specific patterns of expression in roots, stems, young leaves, and mature leaves, suggesting that they may perform different functions in various organs during moso bamboo growth and development. Although the 12 *PeJRLs* examined were expressed in all four tissues, their specific expression patterns differed. The *O. sativa JRL* gene *OsJRL* was also differentially expressed among various tissues, with the highest expression in roots, the lowest expression in leaves, and an intermediate expression level in stems. *OsJRL* acts as a mannose lectin and is mainly found in non-storage tissues such as leaves and roots (Damme et al., 2008). The homologous gene pair *PeJRL06* and -*10* have a similar expression pattern and appear to be root-specific genes, with little expression in stems, young leaves, and mature leaves and higher expression in roots. This suggests that, as homologous genes, their expression is similar, and they may have similar functions.

*JRL* genes are responsive to many different types of hormone induction. For example, *TaJRL1* in wheat responds to SA and MeJA treatments (Xiang et al., 2011). Here, we examined the expression of the *PeJRLs* after treatment with SA, ABA, and MeJA. Four *JRL* genes were upregulated at all times after SA treatment, three genes were upregulated at all times after ABA treatment, and three genes were upregulated at all times after MeJA treatment. However, the expression patterns of different genes from the same subfamily appeared to be different. For instance, *PeJRL08*, *09*, and *13*, all of which have chimeric dirigent-jacalin domains and belong to the same branch of the same subfamily, showed different responses to SA. *PeJRL08* and *PeJRL13* were downregulated or not expressed after 3 h of SA treatment, whereas *PeJRL09* was significantly upregulated. Similarly, *PeJRL08* and *PeJRL24* expression was reduced in response to the three hormone treatments, and *PeJRL07* expression was increased. These results were consistent with those of our previous study in which the dirigent-jacalin chimeric genes *PeD-J02* and *PeD-J03* from moso bamboo were downregulated at different time points after MeJA application (Ma et al., 2021). Here, we again found that *PeJRL09* (*PeD-J02*) and *PeJRL13* (*PeD-J03*) expression was inhibited by MeJA treatment.

The JRL gene expression has been reported to be induced by abiotic stress in many plants, but different gene families exhibit different expression patterns. Here, we analyzed the expression patterns of the *PeJRLs* under low temperature, drought, and salt stresses and combined these results with *cis*-acting element mapping. Most moso bamboo *JRL* genes responded to at least two abiotic stresses. *PeJRL05*, *13*, and *15* were induced by all three stresses, whereas *PeJRL06*, *07*, *08*, *10*, *17*, and *24* were downregulated by all three stresses. This suggests that the *PeJRL* family may function in the stress resistance mechanisms of moso bamboo. In addition, the expression patterns of JRL members from the same subfamily tended to be similar under different stress treatments, with some genes consistently upregulated and other downregulated. This result may be related to the characteristic diversity of expression profiles in the gene family itself, a phenomenon that also occurs in other gene families of moso bamboo (Liu et al., 2017). Interestingly, expression of the homologous gene pair *PeJRL06*/*PeJRL10* decreased under all three stress treatments, suggesting that these genes may function in a similar manner under abiotic stresses. Notably, wheat *TaJRL21*, a homologous chimeric gene with up to 70% similarity to *PeJRL13*, was specifically expressed in leaves, and its expression increased after low temperature and drought stress (Ma et al., 2021). Here, *PeJRL13* expression was highest in stems, and it was upregulated under low temperature, drought, and salt stress conditions. This suggests that *PeJRL13* may perform different functions during the growth and development of moso bamboo. The diverse expression patterns of the *PeJRL* genes reveal that they still play important roles in abiotic stress responses despite their functional differentiation.

### Subcellular Localization and Dimerization Assays

Galactose-binding lectins and mannose-binding lectins are two types of JRL protein (Peumans et al., 2001). Most mannose-binding lectins do not require modification, have no signal peptide, and are localized in the cytoplasm and/or nucleus. In moso bamboo, most PeJRL proteins were hydrophobic, and all but one had no signal peptide (Table 1). Subcellular localization predictions indicated that 13 (52%) PeJRL proteins were localized to the cytoplasm and/or nucleus. We selected *PeJRL13* and *PeJRL04* for transient expression in tobacco and found that they were localized to the nucleus, cell membrane, and cytoplasm (Figure 14B). These results suggest that most *PeJRL* proteins are mannose-binding lectins.

The DJs are chimeric proteins that contain both dirigent and jacalin structural domains, giving them the properties of both gene families and helping them to better facilitate plant stress responses (Chaw et al., 2004). It has been suggested that DJ chimeric proteins are only found in monocots (De Schutter and Van Damme, 2015). In the present study, three-dimensional structural analysis showed that *PeJRL04* contains a typical jacalin domain, whereas *PeJRL13* has both dirigent and jacalin structural domains that form a dimeric structure. The qRT-PCR assays showed that *PeJRL13* expression was significantly upregulated by drought and salt stress (Figures 12, 13), and yeast two-hybrid assays further demonstrated that the chimeric protein *PeJRL13* can interact with itself to form homodimers. Taken together, our results suggest that *PeJRL13* participates in the response of moso bamboo to abiotic stresses in the form of a homodimer.

## Data Availability Statement

The original contributions presented in the study are included in the article/Supplementary Material, further inquiries can be directed to the corresponding authors.

## Author Contributions

ZZ and GQ planned and designed the study. BH performed the data collection and bioinformatics analysis. ZZ wrote the manuscript. JC, YJ, and HG performed the experiments. SL and MR assisted in the interpretation of the results. ZZ, BH, MR, and JC revised and edited the final version of the manuscript. All authors contributed to the article and approved the submitted version.

## Conflict of Interest

The authors declare that the research was conducted in the absence of any commercial or financial relationships that could be construed as a potential conflict of interest.

## Publisher’s Note

All claims expressed in this article are solely those of the authors and do not necessarily represent those of their affiliated organizations, or those of the publisher, the editors and the reviewers. Any product that may be evaluated in this article, or claim that may be made by its manufacturer, is not guaranteed or endorsed by the publisher.

## Footnotes

1
ftp://parrot.genomics.cn/gigadb/pub/10.5524/100001_101000/100498/

2
http://pfam.xfam.org/

3
http://www.hmmer.org/

4
http://smart.embl-heidelberg.de/

5
http://www.ebi.ac.uk/interpro/

6
http://www.csbio.sjtu.edu.cn/bioinf/Cell-PLoc-2/

7
http://www.expasy.org/tools/

8
https://services.healthtech.dtu.dk/service.php?SignalP-5.0

9
http://rice.uga.edu/

10
https://phytozome.jgi.doe.gov

11
http://plants.ensembl.org/index.html

12
www.psc.edu/BioMed/Genedoc

13
https://meme-suite.org/meme/tools/meme

14
https://www.ncbi.nlm.nih.gov/cdd/

15
http://bioinformatics.psb.ugent.be/webtools/plantcare/html/

16
https://github.com/CJ-Chen/TBtools

17
http://github.com/tanghaibao/GOatools

## Supplementary Material

Supplementary Figure 1 Diagram of the *PeJRL* gene family. **(A)** Gene structure diagram. **(B)** Motif analysis. **(C)** Sequence of conserved motifs.

Supplementary Figure 2 Structural domain composition of PeJRL proteins. Two types of structural domains exist for all proteins: type I and type II.

Supplementary Table 1 The specific primers of *PeJRL* genes.

Supplementary Table 2 *K*_a_ and *K*_s_ values for moso bamboo and orthologous (*Pe*-*Os*, *Pe*-*Zm*, and *Pe*-*Bd*) gene pairs.

Supplementary Table 3 Detailed information on gene ontology enrichment analysis of the PeJRL family.

Supplementary Table 4 *JRL* gene IDs and protein sequences from different Gramineae.
