# Supplementary figures and images for: Genome-Wide Identification of JRL Genes in Moso Bamboo and Their Expression Profiles in Response to Multiple Hormones and Abiotic Stresses
Source: Front Plant Sci. 2022 Jan 14;12:809666. doi: 10.3389/fpls.2021.809666 (PMC8795371; doi:10.3389/fpls.2021.809666)

A

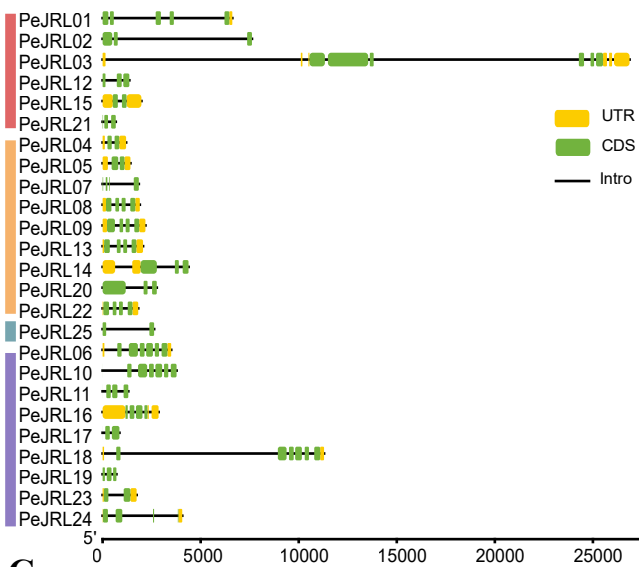

B

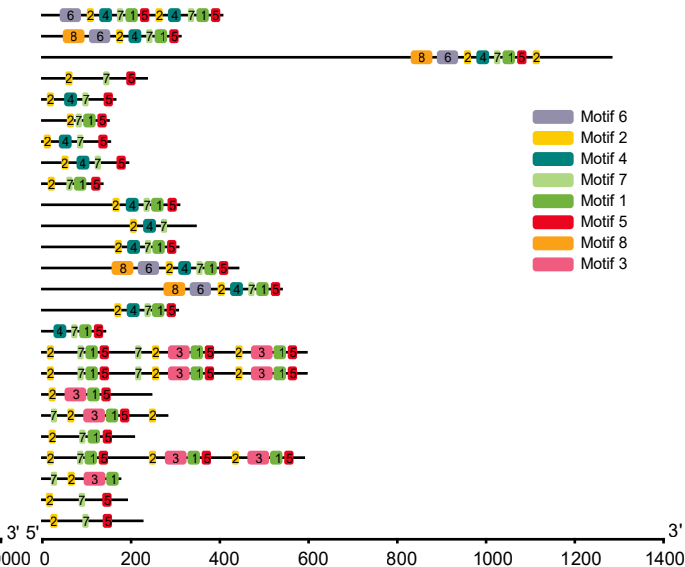

C

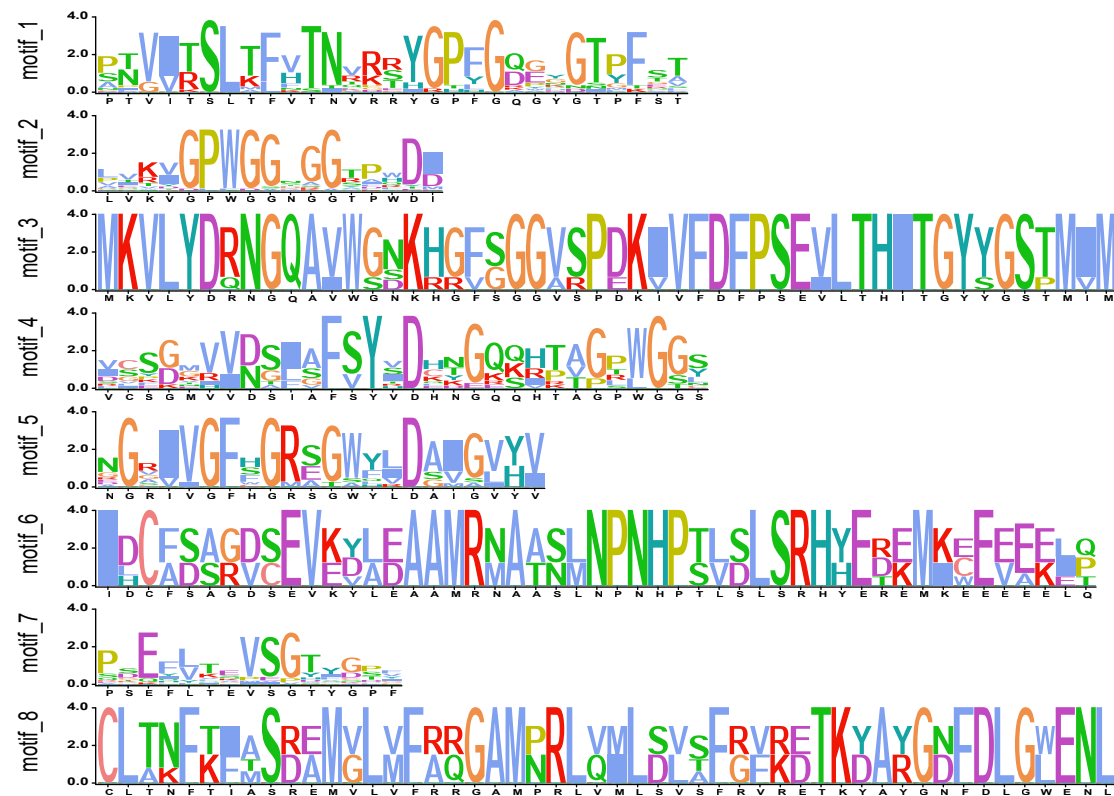

Supplement: Supplementary Figure 1 — Diagram of the PeJRL gene family. (A) Gene structure diagram. (B) Motif analysis. (C) Sequence of conserved motifs. [file Image_1.pdf]

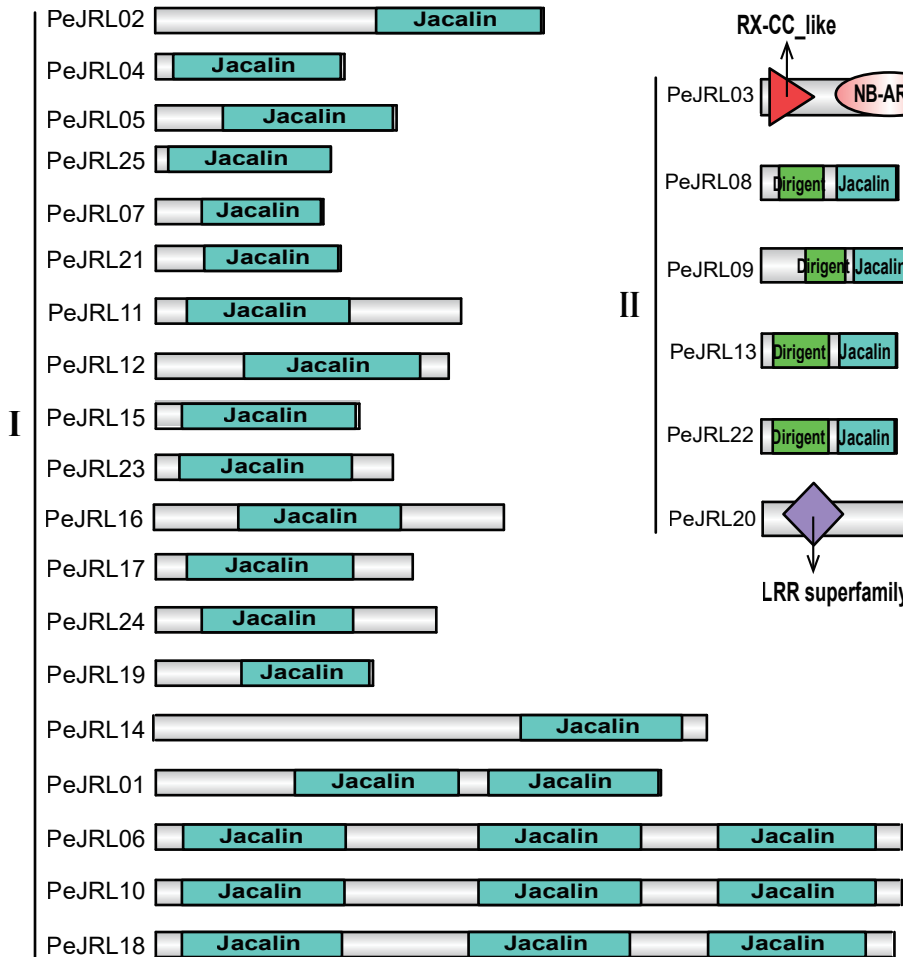

**II**

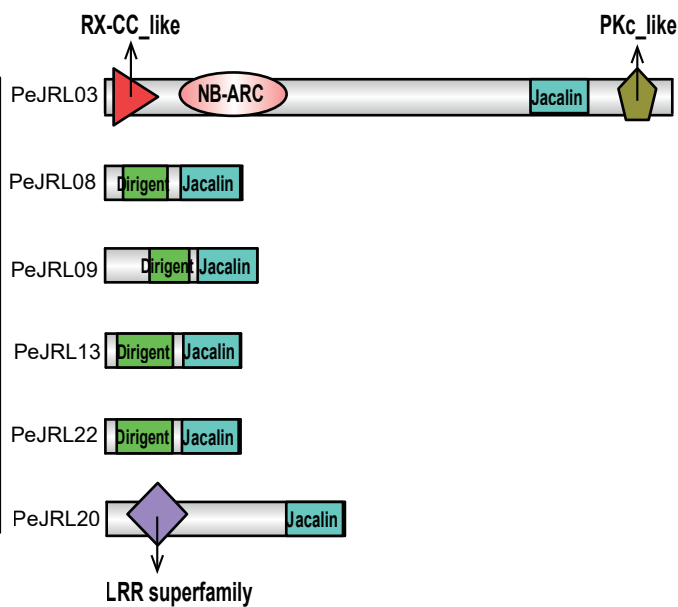

Supplement: Supplementary Figure 2 — Structural domain composition of PeJRL proteins. Two types of structural domains exist for all proteins: type I and type II. [file Image_2.pdf]
